# Biogenic Control of Manganese Doping in Zinc Sulfide Nanomaterial Using *Shewanella oneidensis* MR-1

**DOI:** 10.3389/fmicb.2019.00938

**Published:** 2019-05-07

**Authors:** Prithiviraj Chellamuthu, Kyle Naughton, Sahand Pirbadian, Kalinga Pavan T. Silva, Marko S. Chavez, Mohamed Y. El-Naggar, James Boedicker

**Affiliations:** ^1^Department of Physics and Astronomy, University of Southern California, Los Angeles, CA, United States; ^2^Department of Biological Sciences, University of Southern California, Los Angeles, CA, United States; ^3^Department of Chemistry, University of Southern California, Los Angeles, CA, United States

**Keywords:** *Shewanella*, nanoparticle (NP), genetic engineering, quantum dot (QD), biogenic

## Abstract

Bacteria naturally alter the redox state of many compounds and perform atom-by-atom nanomaterial synthesis to create many inorganic materials. Recent advancements in synthetic biology have spurred interest in using biological systems to manufacture nanomaterials, implementing biological strategies to specify the nanomaterial characteristics such as size, shape, and optical properties. Here, we combine the natural synthetic capabilities of microbes with engineered genetic control circuits toward biogenically synthesized semiconductor nanomaterials. Using an engineered strain of *Shewanella oneindensis* with inducible expression of the cytochrome complex MtrCAB, we control the reduction of manganese (IV) oxide. Cytochrome expression levels were regulated using an inducer molecule, which enabled precise modulation of dopant incorporation into manganese doped zinc sulfide nanoparticles (Mn:ZnS). Thereby, a synthetic gene circuit controlled the optical properties of biogenic quantum dots. These biogenically assembled nanomaterials have similar physical and optoelectronic properties to chemically synthesized particles. Our results demonstrate the promise of implementing synthetic gene circuits for tunable control of nanomaterials made by biological systems.

## Introduction

Modern technology increasingly relies on integrating precision nanomaterials. Indeed, nanomaterials are at the heart of innumerable applications. For applications in catalysis, electrochemistry, biomedical engineering, ultra-strong magnets, and photonics, nanomaterials are a central component in contemporary manufacturing and consumer industries (Vance et al., 2015). To meet the growing demand for nanomaterials in industry and research, fundamentally new methods of synthesis are needed that are both scalable and efficient while at the same time offering the ability to precisely control nanomaterial properties. Current nanomaterial synthesis includes physical, chemical, and biological methods. In biogenic methods, bacteria offer a wide variety of tools to alter the redox state of many compounds and perform atom-by-atom nanomaterial synthesis (Wakatsuki, 1995; Nealson et al., 2002; Rodríguez-Carmona and Villaverde, 2010). By deploying different wild-type bacteria to facilitate nanomaterial growth, researchers may accessdiverse shapes and structures of varying composition, including both inorganic (Au, Ag) and semiconductive (CdTe, ZnS) materials (Naik et al., 2002; Akid et al., 2008; Hussain et al., 2016; Wu and Ng, 2017). Recent advancements in synthetic biology have developed genetic tools to program cellular activity (Ang et al., 2013; Smanski et al., 2016; Schuergers et al., 2017; Segall-Shapiro et al., 2018), including nanomaterial synthesis (Mao et al., 2003; Chen et al., 2014). By combining the natural ability of microbes to assemble nanomaterials under ambient temperature, pressure, and neutral pH with synthetic gene constructs, new routes of nanomaterial synthesis can be developed.

Previous studies developed biogenic routes of nanomaterial synthesis (da Costa et al., 2016; Hussain et al., 2016), including production of chalcogenide nanomaterials such as arsenic sulfide, zinc sulfide, cadmium sulfide, cadmium selenide, and cadmium telluride (Kershaw et al., 2013; Jacob et al., 2016). For example, researchers used a bacteriophage as a template for zinc sulfide nanomaterial nucleation (Mao et al., 2003). In another study, purified proteins were used in direct reduction and nucleation of cadmium sulfide nanomaterials (Dunleavy et al., 2016). Many of these previous studies took advantage of the natural ability of microbes to reduce starting materials and assist in the nucleation and growth of nanomaterials; however, these initial studies did not attempt to integrate natural bacterial biosynthesis with engineered genetic control circuits to tune nanomaterial properties. Recent progress in synthetic biology has developed powerful tools to regulate gene expression and cellular behavior (Spoerke and Voigt, 2007; Smanski et al., 2016; Schuergers et al., 2017). Such gene circuits may enable precise control over the properties of biogenic nanomaterials by tuning the expression level of genetic components involved in the synthesis of inorganic nanomaterials.

Here, a biological system is engineered for the synthesis of zinc sulfide nanomaterials. Zinc sulfur (ZnS) has gathered particular interest in fields of optoelectronic, photocatalytic, and photovoltaic applications (Peng et al., 2006; Son et al., 2007; Stroyuk et al., 2007). Due to a lack of photobleaching, a major drawback observed in fluorescent molecules, ZnS quantum dots have been used in bio-imaging (Deng et al., 2011). Additionally, the lower toxicity levels compared to cadmium sulfide, another common bio-imaging material, further increases the demand for ZnS for medical purposes. Furthermore, doping ZnS enable researchers to engineer a plethora of nanomaterials with different physical properties, sensitively dependent on the dopant level. Doping is a process in which a trace amount of an impurity, often a metal, imparts attractive properties to semiconductor materials (Smith and Nie, 2010). Doping zinc sulfide nanomaterials with manganese (Mn:ZnS) imparts a characteristic emission peak around 590–610 nm, a useful emission wavelength in biological imaging (Deng et al., 2011). Moreover the optical properties of Mn:ZnS nanomaterials are sensitive to the level of doping, and control of the doping level during synthesis is essential. Physical and chemical methods demand high-temperature, high-pH, or high-pressure. Herein, we report a biogenic synthesis route and tunable doping of ZnS:Mn(II) nanomaterials using an anaerobic, metal reducing bacteria *Shewanella oneidensis* at room temperature and pressure.

Using a regulatory genetic circuit, we modulate bacterial electron transfer involved in metal reduction of manganese to synthesize manganese doped zinc sulfide nanomaterials. In an engineered strain of *Shewanella oneidensis* MR-1, a Gram-negative, metal reducing bacteria, the expression of the MtrCAB cytochrome complex was regulated by an external inducer to control the level of manganese doping in ZnS nanomaterials. The properties of the biogenic nanomaterials were similar to nanoparticles used using traditional, chemical synthesis. These results demonstrate the potential for tunable control of the properties of biogenic nanomaterials using synthetic gene circuits.

## Materials and Methods

### Bacteria Culture Conditions

*Shewanella oneidensis* JG3631 strain was obtained from Jeff Gralnick’s lab (University of Minnesota, Minneapolis, MN, United States). The strain has been engineered to express the multi-heme cytochrome complex MtrCAB under control of a native promoter P*torF* that responds to inducer molecule trimethylamine N-oxide (TMAO). Additional information about the strain is available in Supplementary Figure S1. Previously, strain JG3631 reduced iron oxide in proportion to the concentration of TMAO inducer added to the culture. The MtrCAB expression level plateaued at 1,000 μM TMAO (West et al., 2017). Here, we induce cells with 0, 50, 100, and 1,000 μM TMAO. *Shewanella oneidensis* JG1486 was used for control experiments reported in the Supplementary Information, containing deletions of *mtrB*, *mtrE*, *mtrC*, *mtrF*, *mtrA*, *mtrD*, *omcA*, *dmsE*, *SO4360*, *cctA*, and *recA* (Coursolle and Gralnick, 2012).

Cultures of *Shewanella oneidensis* JG3631 were inoculated from a bacterial frozen stock into Luria-Bertani medium and grown overnight (14–16 h) at 30°C under aerobic conditions. Cultures were then transferred to *Shewanella* minimal media prepared from the recipe from Bretschger et al. (2007) with 15 mM lactate as electron donor, 30 mM fumarate as electron acceptor, and TMAO (inducer) at 0, 50, 100, or 1,000 μM. Cultures were grown under anaerobic conditions. After 24 h, the cells grown in minimal medium were centrifuged, washed with 7 mM HEPES buffer, and suspended in 7 mM HEPES buffer to a final OD_600nm_ of 0.8–1.0. Control experiments showed that more dilute cell cultures were also capable of forming ZnS:Mn(II) particles (Supplementary Figure S2). A stoichiometric excess of lactate (10 mM) was used as electron donor in the culture, and the culture was made anaerobic by bubbling sterile nitrogen gas into the bottle. This culture was then used in the experiments for nanomaterial synthesis. Nanomaterial synthesis experiments were performed under anaerobic conditions.

### Biogenic Synthesis of Mn Doped Zinc Sulfide Nanomaterials

Solid manganese (IV) oxide was prepared using the protocol described in an earlier work (Burdige and Nealson, 1985). Prepared manganese (IV) oxide was mixed with HEPES buffer and injected into anaerobic bacterial cultures described above to a final concentration of 750 μM manganese with 10 mM lactate was the electron source. After 24 h of manganese reduction by the bacteria, a filter sterilized stock solution of 2.5 mM zinc sulfate was added to the culture followed by 2.5 mM sodium sulfide. Extended manganese reduction, for 48 h total, did not result in additional manganese reduction or change the photoluminescence of the resulting particles (Supplementary Figure S3). Manganese reduction occurred at 303°K, and samples were moved to room temperature (approximately 295°K) after the addition zinc and sulfide for the remainder of the synthesis reaction. Samples were thoroughly mixed via vortex. The precipitation of nanomaterials started immediately and proceeded for 16 h. Continued manganese reduction was not detected after the addition of zinc sulfate and sodium sulfide, as shown in Supplementary Figure S4. As shown in Supplementary Figure S5, cell viability was maintained throughout the manganese reduction step, however, no live cells were detected 16 h after the addition of zinc sulfate and sodium sulfide.

### Chemical Synthesis of Mn Doped Zinc Sulfide Nanomaterials

Chemical synthesis of Mn:ZnS nanomaterial was accomplished by adding precursors to a sterilized, anaerobic serum bottle containing 25 ml 7 mM HEPES buffer. Sterile nitrogen gas was bubbled through the buffer solution and precursor stock solutions to make them anaerobic. To synthesize Mn:ZnS, first 2.5 mM zinc sulfate was added followed by different manganese acetate at a concentration of either 0, 0.1, 0.5, 1, or 5 mM. Finally, 2.5 mM sodium sulfide was added and the bottle was thoroughly mixed using a vortex. Chemical synthesis was performed at room temperature. The addition of *Shewanella oneidensis* MR-1 cells during chemical synthesis did not appear to impact photoluminescent properties of the chemically synthesized cells, see Supplementary Figure S6.

### Manganese Measurement Using LBB Assay

The reduction of manganese by *Shewanella oneidensis* JG3631 was quantified using the leucoberbelin blue (LBB) assay (Francis et al., 2002). Five hundred microliters was collected directly from well mixed anoxic serum bottles using a sterile, 20G syringe. Sample was added to LBB [0.04% (w/v) LBB in 45 mM acetic acid] to react in the dark for 15–20 min, and then centrifuged to separate the cellular material and insoluble fractions. A standard curve for concentration of was made by preparing serial dilutions of KMnO_4_ and measuring the absorbance at 620 nm to quantify the concentration of Mn. To calculate the amount of Mn(II) at a given time, we subtract the initial amount of Mn(IV) from the amount of Mn(IV) remaining in solution. A calibration curve was made using solutions of potassium permanganate, see Supplementary Figure S7.

### Cleaning and Sonication of Nanomaterial

Upon the completion of nanomaterial synthesis, the contents of the bottle were transferred to a 50 ml conical tube for rinsing and cleaning. Nanomaterial solutions were centrifuged at 3,800 ×*g* for 30 min to collect the nanomaterials, the supernatant was discarded, and the nanomaterial pellet was re-suspended in DI water. This washing step was repeated four times to remove salts and cellular materials from the solution of nanomaterials. In samples where we observed excess aggregation, the final solution of nanomaterials was sonicated in an ice bath for 20 min prior to AFM and SEM.

### Characterization of Nanomaterials Synthesized via Biogenic and Chemical Method

#### Photoluminescence

Nanomaterials synthesized were tested for photoluminescence (PL) emission using a Tecan plate reader (Infinite 200 PRO, excitation wavelength: 325 nm, well mixed condition, 25°C).

#### Absorbance

Nanomaterial samples were mixed and added to a cuvette with 10 mm path length and an absorbance scan was performed using Nanodrop 2000C. Background from media/buffer was subtracted.

#### Scanning Electron Micrograph

Cleaned nanomaterials were deposited on a silicon wafer for electron microscopy and samples were sputter coated (Cressington 108C) with gold. JEOL 7000 electron microscopy was used to image the nanomaterials and EDX was used to characterize the elemental composition of the nanomaterials.

#### Atomic Force Microscopy

Cleaned nanomaterials were diluted to low concentrations in DI water and deposited on graphite substrate for atomic force microscopy (AFM) imaging. Samples were imaged in AC mode in air using an Asylum Cypher ES instrument and an AC mode tip (Asylum Research, silicon probe model AC240TS-R3 with 2 N/m nominal spring constant). The images acquired were analyzed using Gwyddion software. A minimum of 100 individual nanomaterials per sample were analyzed for size calculation.

#### X-Ray Diffraction

Concentrated samples were deposited on a glass slide and used for XRD analysis. The X-ray diffraction (XRD) scattering profiles were obtained using a Rigaku Ultima IV Diffractometer using characteristic Cu Kα radiation = 1.54 Å.

#### EPMA

Concentrated samples were deposited on a silicon substrate for a quantitative elemental analysis using JEOL 8200 electron microprobe (20 kV, focused beam mode). Reference materials used were zinc sulfide and manganese sulfide (Sigma-Aldrich). A minimum of 10 spots were analyzed for quantification of manganese concentration in the zinc sulfide nanoparticles. “Spots” are usually aggregates of nanomaterials, since the sample preparation and deposition resulted in nanomaterials aggregates, therefore individual spots were composed of nanomaterial aggregates.

## Results and Discussion

### Controlling the Dopant Concentration of Mn:ZnS Using a Genetic Circuit

**FIGURE 1:**
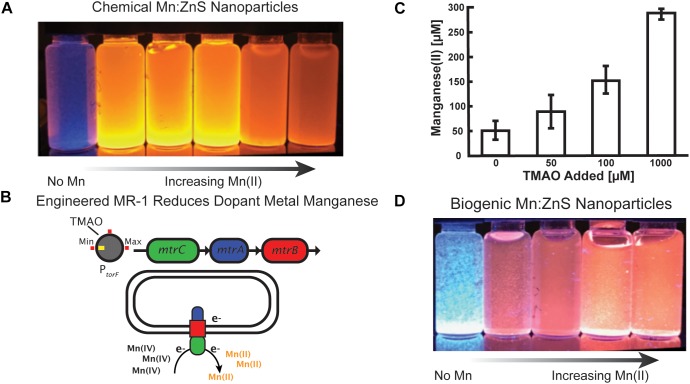
Controlling manganese doping of zinc sulfide nanomaterials by engineered *Shewanella oneidensis* JG3631. **(A)** Chemical synthesis of Mn doped zinc sulfide nanoparticles, demonstrating that increased Mn doping alters the optical properties of the nanoparticles. **(B)** In engineered *Shewanella* MR-1, the TMAO inducer molecule regulates expression of the metal reducing cytochrome complex MtrCAB. MtrCAB performs extracellular reduction of metals. **(C)** Cultures of the engineered *Shewanella* produced Mn(II) concentrations proportional to the concentration of the TMAO inducer. Samples were collected from well mixed cell solutions following 24 h of manganese reduction, and Mn(II) concentrations were measured using the LBB assay. **(D)** Mn doped zinc sulfide nanoparticles synthesized by cells show a change in optical properties as a function of added inducer.

By utilizing a genetically engineered bacteria, we designed a biological system to synthesize semi-conductive ZnS nanomaterials doped with Mn(II). Moreover, the degree to which the gene circuit responds to an outside signal modulates the concentration of available Mn(II) for doping in ZnS, thereby adjusting the optoelectronic properties of these biogenically fabricated nanomaterials. Estimated manganese concentrations using EPMA are presented in Supplementary Table S1. These nanomaterials of biogenic origin exhibit almost identical properties to those made by non-biological, chemical methods. To begin our study we synthesized ZnS nanoparticles via chemical means. In bulk, these particles exhibited a characteristic blue emission upon excitation with UV light (Figure 1A). Next, we introduced variable concentrations of Mn(II) during chemical synthesis and the soluble Mn(II) was passively integrated into the ZnS nanoparticles. These doped nanoparticles exhibited a characteristic orange hue upon excitation with UV light (Figure 1A) (Beerman, 2005; Cao et al., 2009; Deng et al., 2011). The photoluminescent intensity of the Mn(II) doped nanoparticles depended on the level of doping.

To control the optical properties of Mn:ZnS nanoparticles through biogenic route, we used an engineered strain (JG3631) of *Shewanella oneidensis* MR-1 because of its metabolic versatility, whole genome sequence availability, and a library of characterized, engineered strains (Bouhenni et al., 2005; Nealson, 2005; Bretschger et al., 2007; Fredrickson et al., 2008). *Shewanella* naturally respire insoluble metal oxides of iron and manganese via extracellular electron transport protein complex MtrCAB, a multiheme cytochrome complex. This protein complex moves electrons from the periplasmic space to the exterior of the cell during respiration (Myers and Myers, 2001; Nealson et al., 2002; Bretschger et al., 2007). Expressed under anaerobic conditions, the Mtr pathway is composed of three components: MtrA, a periplasmic decaheme c-cytochrome; MtrB, an outer membrane porin; and MtrC, an outer membrane decaheme c-type cytochrome. Here, we utilize an engineered strain JG3631 in which an inducible promotor P*torF* regulates expression of the *mtrCAB* operon (Figure 1B) (West et al., 2017). Previously it was shown that this strain reduced external iron oxide in proportion to the amount of the inducer (TMAO) added to the culture (West et al., 2017).

First, we tested the ability of the strain JG3631 to reduce manganese in the presence of different TMAO concentrations. As shown in Figure 1C, the amount of Mn(IV) reduced by the cell culture was proportional to the concentration of inducer molecule TMAO. However, at 0 mM TMAO, some manganese reduction was observed, potentially due to a combination of leaky expression of *mtrCAB* and additional biochemical pathways involved in low levels of manganese reduction, such as MtrDEF cytochrome (Coursolle and Gralnick, 2010). Additional information on the engineered strain and the experimental outline is presented in the Supplementary Figure S1.

After confirming that we could control manganese reduction, and therefore the available concentration of Mn(II) via the concentration of TMAO inducer molecule, we investigated the biogenic synthesis of Mn:ZnS. Because *Shewanella* prefer to express metal reduction protein complexes in the absence of oxygen, all biogenic reactions took place under anaerobic conditions, as outlined in the “Materials and Methods” section. The nanoparticles produced without Mn(IV) added to the culture appear blue under UV excitation (Figure 1D, left). By adding TMAO, however, the MtrCAB pathway is modulated in a manner reflecting the concentration of inducer molecule (Figure 1C), i.e., more TMAO results in more insoluble Mn(IV) being reduced to soluble Mn(II). As expected, nanoparticles produced in the presence of both Mn(IV) and TMAO exhibit a characteristic red-shifted emission upon UV excitation, similar to chemical synthesis. The buffer solutions and the precursor solutions did not exhibit any UV associated luminescence (Supplementary Figure S8). Altogether, by connecting a naturally occurring metal reduction route in an engineered strain of *Shewanella* with an inducible promotor, we made possible the controllable synthesis of nanoparticles with tunable optoelectronic properties.

### Optoelectronic Properties of ZnS and Mn:ZnS Nanomaterials

**FIGURE 2:**
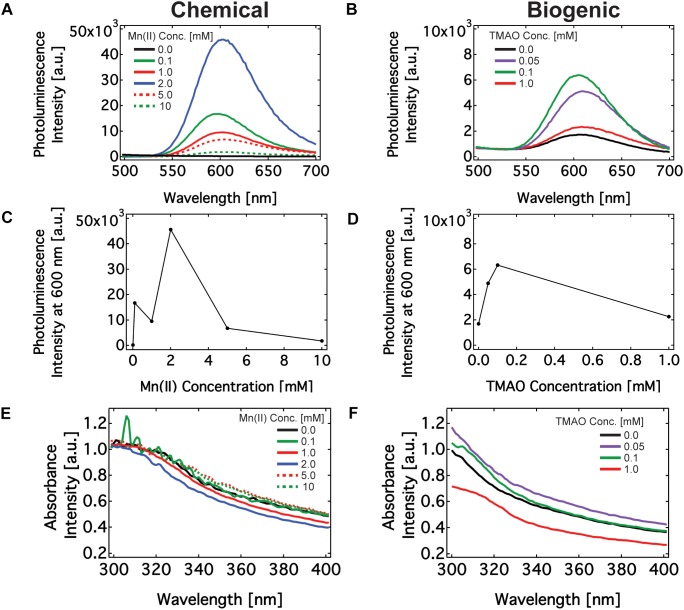
Optical characterization of Mn doped ZnS nanomaterials synthesized via chemical route and biogenic route using engineered *Shewanella oneidensis* MR-1. **(A,B)** PL emission spectrum of nanomaterials excited at 325 nm made using chemical **(A)** and biogenic **(B)** methods. **(C,D)** The maximum PL emission intensity of nanomaterials made using chemical **(C)** and biogenic **(D)** methods. **(E,F)** Absorbance spectrum of nanomaterials made using chemical **(E)** and biogenic **(F)** methods.

Following chemical and biogenic synthesis, we assessed the optoelectronic properties (e.g., photoluminescence and absorbance) of the Mn:ZnS. Both methods yielded nanomaterials with the characteristic 600 nm photoluminescence (PL) peak associated with the presence of dopant metal Mn(II) in a ZnS lattice. The shift in PL emission due to Mn(II) doping into the ZnS nanomaterial is caused by an additional electronic transition between excited electrons and the energy levels of the Mn dopant (Bhargava et al., 1994; Karar et al., 2004). Chemically synthesized nanomaterials produced PL peaks in the 602–604 nm range and the biogenic nanomaterials produced peaks in the 604–608 nm range (Figure 2A,B). The difference in the PL peak emission between the two methods may be a result of biogenic moieties altering the emission spectrum. Earlier work showed that PL emission wavelength of ZnS nanoparticles varied according to the capping agent used during the reaction (Warad et al., 2005; Wanjari et al., 2015).

Buffers, and precursor solutions did not produce any absorbance or PL emission peaks (Supplementary Figure S8). Manganese may react with sulfide to produce manganese sulfide (MnS) precipitate. Some groups have observed PL emission on MnS films near 640 nm, which could explain the slight red shift in the PL peak with increasing Mn(II) concentration (Goede et al., 1986). We do not observe any PL optical signature of MnS nanoparticles in this region (Supplementary Figure S8). This finding leaves Mn:ZnS as the only candidate emitter at 600 nm during PL characterization. Next, we examined the effect of biologically regulated Mn(II) concentration on the PL emission of the nanoparticles. In the chemical method, the PL intensity increased up to 2 mM Mn(II) and subsequently decreased. In the biogenic method, the PL intensity increased up to 100 μM TMAO induction and then decreased with increasing concentrations of TMAO. Recall that TMAO concentrations dictates the concentrations of soluble Mn(II). This observed effect of dopant concentrations on PL emission peak intensity — an increase followed by a decrease upon reaching a critical concentration (Figure 2C,D) — is consistent with previous results (Son et al., 2007; Cao et al., 2009). This effect, known as luminesce quenching, is a common feature among doped semi-conductors, of which Mn(II) doped ZnS is a classic example (Hurd and King, 1979; Sasakura et al., 1981; Katiyar and Kitai, 1992). Briefly, the probability of an electron indirectly transitioning through a Mn(II) site to the ground state increases with Mn concentration up to 1–4 wt%. As the Mn concentration increases beyond a few percent by weight, however, unfavorable interactions between adjacent Mn(II) sites, interruptions in the ZnS crystallinity, an increase in non-radiative transition processes, and a decrease the in the excitation efficiency from the ZnS lattice diminish the effect of doping. Finally, it is worth noting that where the Mn(II) ions subsume with the nanomaterial dramatically affects its optoelectronic properties. It is known that incorporating Mn(II) on the surface of ZnS quantum dots instead of the bulk results in an ultraviolet PL emission. That our nanomaterial emits in the visible region near 600 nm suggests that the majority of Mn(II) is embedded within the ZnS structure rather than the surface (Xiao and Xiao, 2008). The PL emission intensity was generally higher in chemically synthesized particles, and it is currently unclear if synthesis in the presence of cellular compounds impacts the intensity of the PL emission. Overall, the PL properties of the chemically and biogenically synthesized Mn:ZnS nanomaterials are similar to each other and with literature precedent.

Next, we measured the absorption spectrum for biogenically and chemically synthesized nanomaterials in the 300–700 nm wavelength windows. Figure 2E,F show the absorption spectrum for the nanoparticles is in the UV wavelength regions of 310–320 nm, which agrees with earlier work (Kole and Kumbhakar, 2012). Chemically synthesized nanoparticles had an absorption peak of 310 nm and the biogenic nanoparticles had an absorption peak of 315 nm. From the absorbance, one may find the band gap of the nanomaterials. The band gap energies of nanomaterials made by chemical and biogenic methods are 4.0 eV and 3.9 eV respectively. These values are higher than for bulk ZnS material, which is 3.7 eV. An inverse relationship exists between the size of the nanoparticle and its bandgap due to quantum mechanical confinement, i.e., the system resembles a particle-in-a-box. The bandgap is the energetic difference between the ground state and excited state (the bands) of the electron in an atom or bulk material. A single atom possesses a large bandgap because the allowed electron states are precisely defined. That is, the band is extremely narrow and the gap between bands (the bandgap) is large. A continuous, bulk material, however, is composed of many, overlapping electron orbitals. The effect of overlapping increases the band width with a concomitant decrease between the ground and excited state of electrons in the material. As a result, the band is wider and the gap between bands decreases (Son et al., 2007; Begum and Chattopadhyay, 2014; Marusak et al., 2016).

### Crystalline Structure Analysis of ZnS and Mn:ZnS Nanomaterials

**FIGURE 3:**
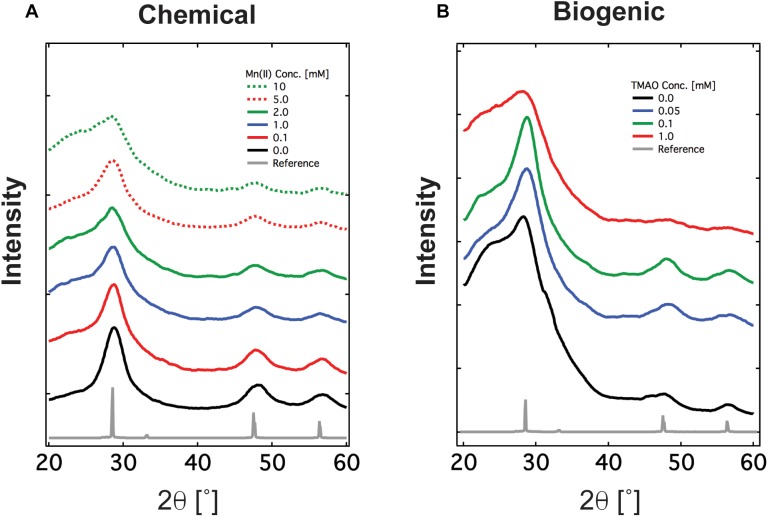
XRD spectrum of Mn doped ZnS nanomaterials synthesized via **(A)** the chemical route and **(B)** the biogenic route using engineered *Shewanella oneidensis* JG3631. XRD spectrum confirms the material synthesized is crystalline and has zincblende crystalline orientation, shown as reference.

Next, we characterized the crystalline structure of Mn:ZnS synthesized by the chemical and biogenic methods via XRD, an X-ray scattering technique which measures coherent diffraction from crystalline domains within nanomaterials across an entire sample. The XRD pattern indicated there were three distinct diffraction peaks (28.7°, 47.9°, and 56.7°) with 2𝜃 values corresponding to three planes (111), (220), and (311). These peaks confirm that the synthesized nanomaterial has a cubic phase of zinc blende consistent with previous reports and zinc sulfide standard (Supplementary Figure S9). By analyzing the XRD data using the Scherrer model (Supplementary Equation S1), one may extract the size of the crystalline domains within the nanomaterials. Briefly, the width of the diffraction peak is inversely proportional to the size of the crystalline domain. For both the chemically and biogenically synthesized nanomaterials, the crystalline domain is between 5 and 8 nm in diameter (Supplementary Table S2), slightly smaller than the particle size derived from optical methods. This is not unexpected, as the geometry and crystallinity of nanoparticles may differ due to the presence of amorphous, X-ray diffusive regions, which XRD cannot detect. Finally, as further evidence that ZnS and Mn:ZnS made the bulk of the nanomaterial, no peaks associated with MnS crystals appear in the XRD pattern of our purified nanomaterials (Figure 3 and Supplementary Figure S9). To determine if the presence of Mn(IV) during synthesis influenced nanoparticle properties, Mn(IV) oxide was added during chemical synthesis. The addition of Mn(IV) oxide did not inhibit the formation of ZnS and ZnS:Mn(II) nanoparticles, see Supplementary Figure S9B. Also, Mn(IV) oxide alone was not sufficient for manganese doping during chemical synthesis, see Supplementary Figure S10. These results are in line with earlier work and show good agreement between the chemical and biogenic route (Son et al., 2007).

### Morphology, Elemental Analysis, and Size Distribution of ZnS and Mn:ZnS

Next, we leveraged energy-dispersive X-ray spectroscopy (EDX) measurements to confirm the elemental composition of the nanomaterial. In an EDX experiment, X-ray excitation induces emission spectra unique to specific atomic nuclei in the material, thereby furnishing information about the specific elemental make-up of the sample. The peaks of zinc and sulfur indicate the material formed was zinc sulfide (Supplementary Figures S11, S12) in agreement with the XRD data. The primary peaks in these data correspond to Si and O and likely arise from the sample substrate, a Si_2_ wafer. The secondary peaks, however, correspond to Zn, S, and Mn (in doped samples). It is worth noting that the peaks are unlikely from left over substrates as the samples were thoroughly washed to remove the initial reactants used for nanomaterial synthesis.

**FIGURE 4:**
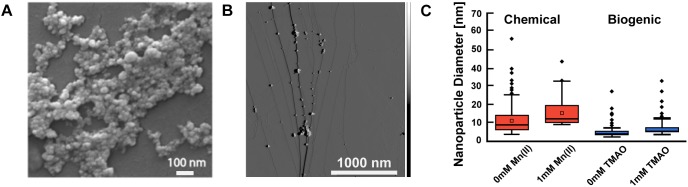
Electron microscopy and atomic force microscopy characterization of biogenic Mn doped zinc sulfide nanomaterials. **(A)** SEM image of Mn doped zinc sulfide nanomaterials (chemical synthesis 1% Mn). **(B)** AFM image of biogenic Mn doped zinc sulfide nanomaterials. **(C)** Sizes of nanomaterials synthesized via chemical method and biogenic method using AFM. A minumum of 150 nanoparticles were analyzed (*n* > 150); particle clusters were omitted. The box indicates the interquartile range, which captures all the data between the first and third quartile. The horizontal line within the box represents the mean or expected value. The bars extending above and below indicate the min and max of the data, excluding outliers. The dots outside the min and max lines represent outliers, which are values 1.5 inner quartile range distance from the inner quartile. The box plot represents data from one replicate of each sample. Comparison of particle sizes using an unpaired Student’s *t*-test revealed that all pairs of measurements were significantly different (*p* < 0.01) except for biogenic particles at 0 and 1 mM TMAO which had a *p* > 0.05.

Next, we used scanning electron microscopy (SEM) and AFM to identify the size and shape of the synthesized ZnS and Mn:ZnS nanomaterials. SEM images showed that particles were quasi-spherical (Figure 4A) in good agreement with earlier work (Warad et al., 2005; Chandrakar et al., 2015). SEM analysis was used for identifying the morphology of the nanoparticles, while AFM was used to quantify the size of the nanoparticles.

We measured the size of the ZnS and Mn:ZnS nanomaterials using AFM. AFM size analysis (Figure 4B,C) reveals that the size of Mn:ZnS nanomaterials synthesized via chemical method was slightly larger and polydisperse compared to nanomaterials synthesized via biogenic method. Researchers have measured the size of chemically synthesized Mn:ZnS nanomaterials in the range of 2–70 nm (Warad et al., 2005; Cao et al., 2009; Kole and Kumbhakar, 2012; Komada et al., 2012). The mean value for the chemically synthesized particles is 8.7 nm [0 mM Mn(II)], 11.5 nm [1 mM Mn(II)] and the mean value for the biogenically synthesized particles is 4.0 nm (0 TMAO, no induction) and 5.0 nm (1 mM TMAO, full induction). The difference in size and polydisperity may result from different reaction kinetics between the chemical and biogenic methods, an advantage in nanomaterial sysnthesis that merits further investigating. Altogether, EDX, SEM, and AFM reveal that the nanomaterials synthesized via chemical and biogenic routes were pure, spherical, and approximately 2–20 nm.

## Conclusion

We synthesized manganese doped zinc sulfide (Mn:ZnS) nanoparticles using chemical and biogenic methods. Mn:ZnS nanoparticles have applications as field emission materials, field effect transistors (FETs), p-type conductors, biosensors, chemical sensors, and catalysts, and nanogenerator (Fang et al., 2011). Previous studies have shown that biogenic Mn:ZnS nanoparticles exhibit biocompatibility and lesser toxicity in biomedical imaging applications as compared to chemically synthesized nanoparticles (Hazra et al., 2013). Here, both methods yielded nanoparticles with a characteristic PL emission peak at 600 nm, although in general the PL emission spectrum of Mn:ZnS nanomaterials is known to vary with synthesis method (Cao et al., 2009; Deng et al., 2011; Komada et al., 2012; Ali et al., 2016).

To optimize the biogenic synthesis process, experimental design should consider the location of nanoparticle synthesis. For example, nanomaterials produced by bacteria may be nucleated and grown in the cytoplasmic, periplasmic, or extracellular space. These three regions, especially the cytoplasm and periplasm, contain a variety of biomolecular moieties, each of which influence synthesis. Moreover, harvesting nanomaterials from the interior of the cell may require lysing cells, which can introduce additional post-production modifications to the nanomaterials, like capping. Designing biogenetic nanomaterial synthesis routes with optimal or minimal biomolecular blends should therefore consider focusing nanomaterials synthesis on/outside the cell. Following this line of reasoning Marusak et al. (2016) explored the relationship between stages of bacterial cell growth of *E. coli* on the synthesis of the CdS nanoparticles. By adding CdCl_2_ to *E. coli* cultures after an initial 10-h growth period, CdS nanomaterial formation was largely extracellular, which reduced doping by non-specific agents. Likewise, in our system, because the MtrCAB protein complex extends from the periplasmic to extracellular space, we suspect that ZnS and Mn:ZnS nanomaterials originate outside the cell. Subsequent work will explore the origin and control of nanomaterial nucleation/growth.

Although this work focuses on Mn(II) as a dopant, the feasibility of incorporating additional dopant(s) through biogenic routes should be investigated. Other groups have reported chemically synthesizing ZnS with dopants as varied as nickel, cadmium, and copper (Biswas et al., 2006; Fang et al., 2011). Although the cell culture itself is reducing, additional control experiments shown in Supplementary Figure S13 show that *mtr* expression and activity were needed for manganese doping. Removal of the carbon source during the manganese reduction step was not sufficient to completely eliminate the reducing activity of cells expressing *mtr*. Future work should look at the wide variety of available cytochromes and their capability to reduce many transition metals, lanthanides, and actinides (Lloyd, 2003). These starting materials could also be incorporated into biogenically derived nanomaterials.

As a complement to doping, tuning the size of the nanomaterial offers a parallel and complementary level of control over the optoelectronic properties. Particle size of the manganese doped nanoparticles zinc sulfide slightly varies according to the synthesis method, which is not unexpected since each method will have specific nucleating factors and reaction kinetics which influence the size and growth of the nanomaterials. Capping agents from various sources such as plants, fungi, and bacteria cells have been used to control the size and nucleation of nanomaterials (Singh et al., 2011; Wanjari et al., 2015; Hussain et al., 2016). In our work, cellular biomolecules in the exo-, peri-, or cytoplasm may have played a role in determining the size and geometry of nanomaterials. For example, biofilm surfactins of many microbes are known to alter nanoparticle size (Singh et al., 2011, 2014; Rodrigues, 2015). As in controlled Mn doping, synthetic gene circuits regulating genes involved in production and secretion of biological capping agents could control nanomaterial morphology. Such biological control over multiple facets of nanoparticle synthesis may produce nanomaterials unavailable via chemical methods. Realizing the potential of biogenic nanomaterial synthesis will benefit from future developments in both synthetic gene circuits and an increased understanding of how microbes influence nanomaterial formation.

## Author Contributions

PC, KN, KS, JB, and ME-N contributed to the conception and design of the study. SP and MC performed some characterization and experiments. PC wrote the first draft of the manuscript. PC, KN, and JB wrote sections of the manuscript. All authors contributed to manuscript revision, read and approved the submitted version.

## Conflict of Interest Statement

The authors declare that the research was conducted in the absence of any commercial or financial relationships that could be construed as a potential conflict of interest.
