# Supplementary material for: Biogenic Control of Manganese Doping in Zinc Sulfide Nanomaterial Using Shewanella oneidensis MR-1
Source: Front Microbiol. 2019 May 7;10:938. doi: 10.3389/fmicb.2019.00938 (PMC6514046; doi:10.3389/fmicb.2019.00938)
Supplement: Supplementary file 1 [file Data_Sheet_1.pdf]

Supplementary Material

Engineered MR-1 Strain Information

Shewanella oneidensis MR-1 is a metal reducing bacterium known to reduce various metals such as iron, manganese, chromium, selenium, and sulfur. MR-1 possesses numerous cytochromes that gives the organism the ability to perform diverse electron transfer capability. Among the cytochromes, MtrCAB is a well-characterized decaheme cytochrome involved in transferring electrons to solid metal oxides. All three proteins MtrA, MtrB, MtrC are necessary to form a functional cytochrome. In the WT strain, it is expressed under anaerobic conditions regulated by the native promoter p*mtr*CAB. In order to control the expression levels of MtrCAB, Gralnick et al, engineered a strain by removing native promoter (P*mtrCAB*) and replacing it with an inducible promoter (P*torF*) that responds to TMAO concentration levels. The promoter (PtorF) is an indigenous promoter native to the MR-1 WT strain; it controls the expression levels of TMAO reductase genes, involved in the respiration of TMAO. TMAO could be metabolized by *Shewanella*; to avoid the inducer molecule being used by the organism, the researchers deleted the TMAO reductase gene. JG3631 strain had deletion of Δ*torEFADF* (TMAO respiration), and Δ*dmsEFABGH (*DMSO respiration). The strain has been characterized for extracellular electron transport in a microbial fuel cell and iron oxide reduction(West *et al.*, 2017)**.**

A)

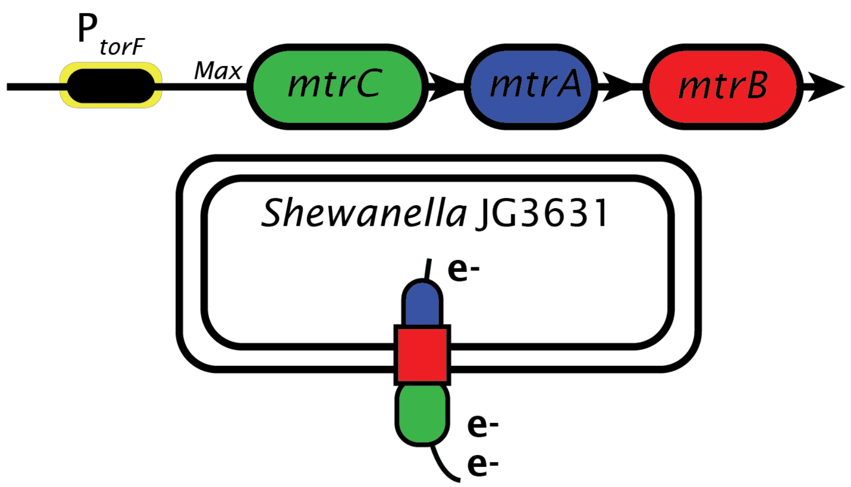

B)

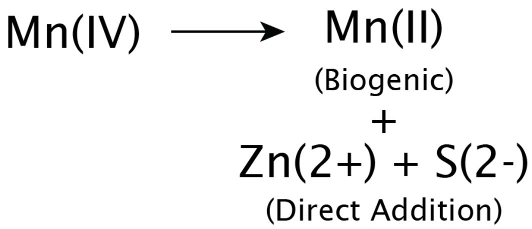

Figure S1: Tunable genetic circuit of JG3631 enables to control the dopant concentration of Mn(II) in Mn:ZnS nanoparticles. A) JG3631 strain has P*torF* that is inducible with TMAO in the place of native promoter P*mtrCAB*. MtrCAB cytochrome is involved in extracellular electron transfer to insoluble manganese oxide; increasing the expression of MtrCAB increases the reduction of manganese (IV) oxide to manganese (II) oxide used as the dopant in the ZnS nanomaterials. By increasing the concentration of TMAO, we saw an increase in Mn(IV) reduction through increased expression of MtrCAB cytochromes B) Nanomaterial synthesis using biogenic Mn(II) with direct addition of zinc and sulfide.

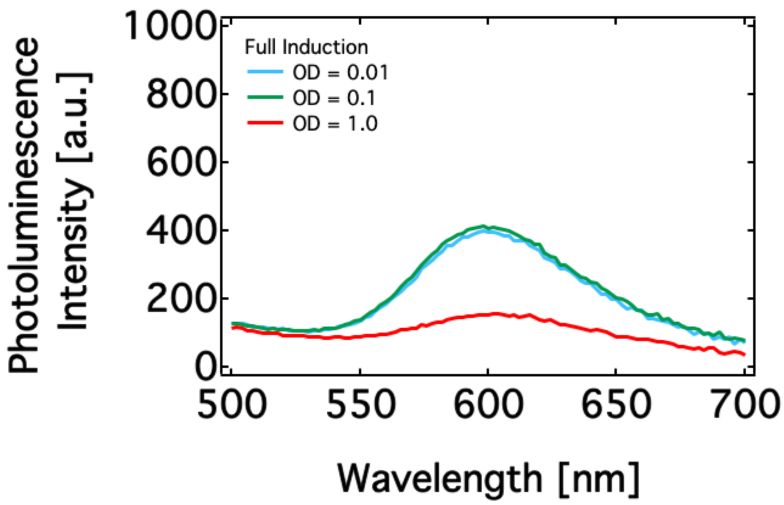

Figure S2: The influence of cell density on manganese doping. Cell density was adjusted to an OD_{600} measured at 1 cm path length of 0.01, 0.1 and 1.0. prior to the addition of Mn(IV). Cultures were *Shewanella oneidensis* JG3631 induced with 1 mM of TMAO. The photoluminescence spectrum of the resulting ZnS:Mn(II) particles is shown, indicating manganese doping at all cell densities.

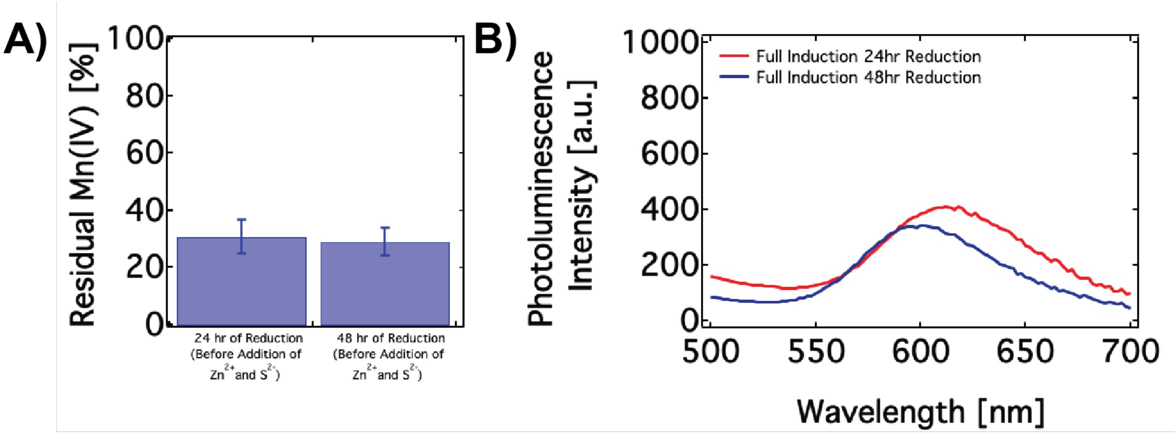

Figure S3: The influence of the manganese reduction time on manganese doping. (A) Amount of Mn(IV) in solution was measured for a culture of *Shewanella oneidensis* JG3631 induced with 1 mM of TMAO after both 24 h (the typical time used our experiments) and 48 hours using the LBB assay. Result indicate the additional 24 h of reduction resulted in no significant changes in manganese reduction. (B) Photoluminescence spectrum of ZnS:Mn(II) particles using the standard 24 h manganese reduction step and the 48 h manganese reduction step.

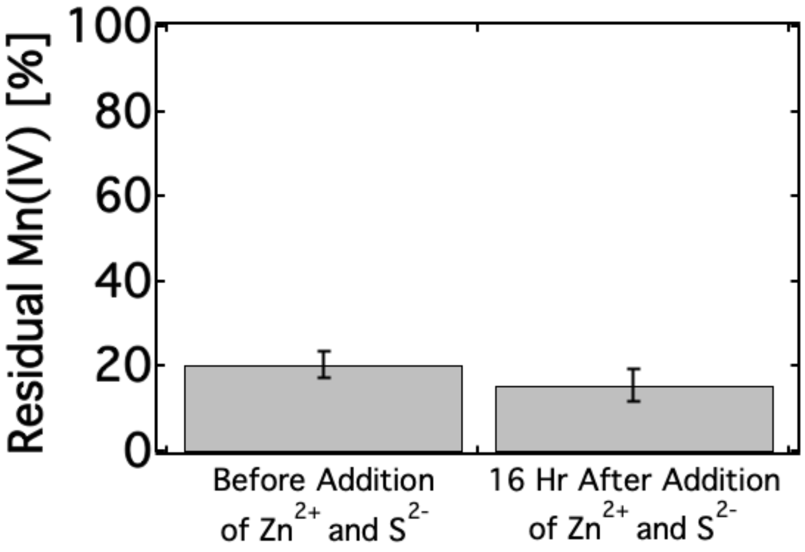

Figure S4: Mn(IV) reduction after addition of zinc sulfate and sodium sulfide. The amount of Mn(IV) in the cell solution was measured before and 16 hours after the addition of zinc sulfate and sodium sulfide using the LBB assay. Experiments were run with *Shewanella oneidensis* JG3631 fully induced with 1 mM TMAO. Error bars show standard deviation of three measurements.

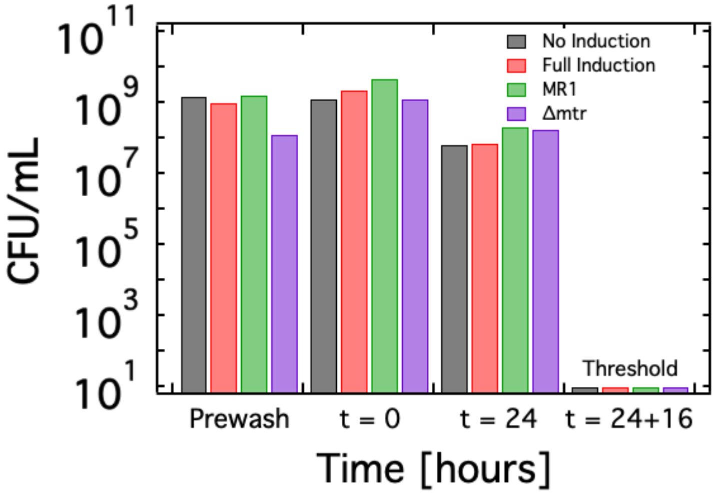

Figure S5: Viability of cells during nanoparticle synthesis. At t=0, cells were resuspended in media with lactate and Mn(IV) and adjusted to OD_{600} = 1.0. Mn(IV) reduction continued for 24 hours. At t = 24 h, Zn(II) and sulfide were added to the media, and nanoparticle synthesis continued for 16 hours. Cell viability was accessed via plate counting on LB plates. Strains tested were *Shewanella oneidensis* MR-1 (MR1), *Shewanella oneidensis* JG3631 at both 0 and 1 mM TMAO (no induction and full induction respectively), and a *Shewanella oneidensis* JG1486 with deletion of the *mtr* pathway (Δ*mtr*).

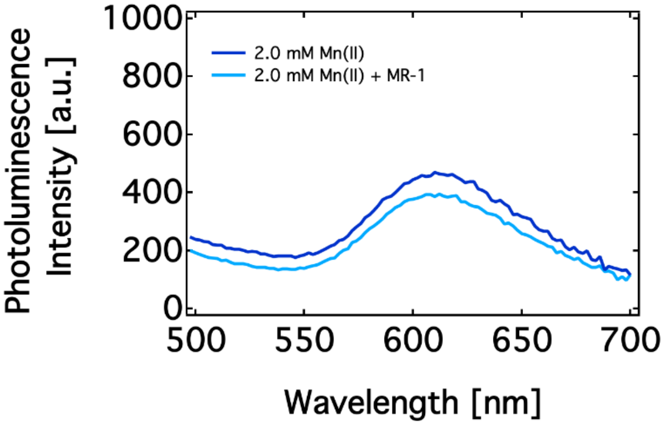

Figure S6: The impact of cells on the synthesis of ZnS:Mn(II) particles. *Shewanella oneidensis* MR-1 cells, at an OD_{600} of 1.0 were added during chemical synthesis. Photoluminescence spectrum of cells chemically synthesized in the presence and absence of cells is shown.

Manganese reduction

Manganese reduction was measured using Leucoberbelin (LBB) assay. Manganese with higher oxidation states (+7, +4, and +3) reacts with LBB reagent to produced colored solution, whose absorbance was measured at 620nm. The concentration of manganese oxide (Mn(IV)) is directly proportional to the absorbance at 620 nm. *Shewanella* reduces Mn(IV) to Mn(II), and the absorbance will go down over time with increased cellular reduction of manganese. Standard curve was created using potassium permanganate (Mn(VII)); 40 µM $KMnO_4$ is equivalent to 100 µM MnO_2. In our experiments, 0.1 ml sample was collected and mixed with 0.9 ml of LBB solution. Following a 15 minutes incubation in the dark that is necessary for the completion of the reaction, we measured Abs_{620nm}. Concentrations of Mn(IV) in the samples calculated from the calibration curve.

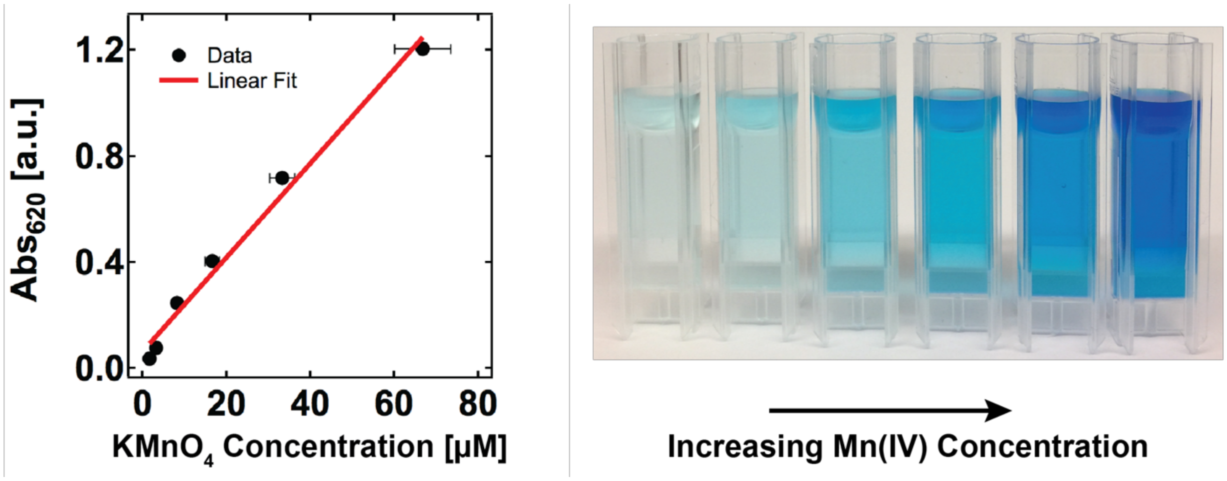

Figure S7: Standard curve for manganese oxide measurement using Leucoberbelin (LBB) assay. *Shewanella* reduces $Mn0_2$ (Mn(IV)) to MnO (Mn(II)). Equivalent concentrations of Mn(IV) were calculated from the standard curve (40 µM $KMnO_4$ is equivalent to 100 µM MnO_2).

Condition	Mn(II) [μM]*	Incorporated Mn(II) [μM]	
		Mean	Std. Dev
Abiotic	0	0	0.02
Abiotic	100	8.3	0.12
Abiotic	1000	15.9	0.14
Abiotic	2000	26.7	0.16
Abiotic	5000	19.8	0.56
Biogenic 0μM TMAO	50	2.3	0.07
Biogenic 50μM TMAO	95	5.4	0.16
Biogenic 100μM TMAO	148	12.3	0.12
Biogenic 1000 μM TMAO	293	10.8	0.16

Table S1: Percentage of manganese incorporated from the reaction into the zinc sulfide nanoparticles. The concentrations are averages of nanoparticle cluster and not individual nanoparticles. In biogenic synthesis method, TMAO molecule controls the concentration of manganese in the ZnS nanoparticles. Incorporated Mn(II) measured by EPMA. *For biogenic condition, the available Mn(II) in solution was calculated from curve in Fig S2.

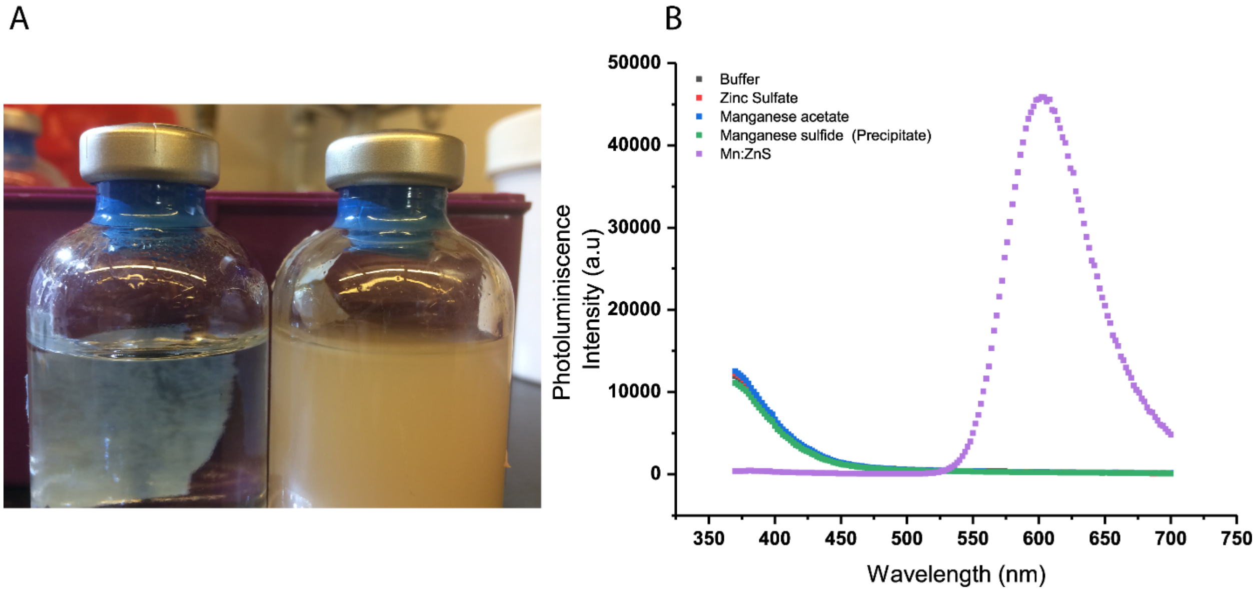

Figure S8: PL emission at 600 nm is characteristic of Mn:ZnS. Abiotic controls do not produce PL emission. A) Control reactions of buffer and bottle with manganese and sulfide precipitate; B) Manganese sulfide precipitate, buffer, zinc sulfate, and manganese acetate does not produce any emission spectrum when excited at 325 nm.

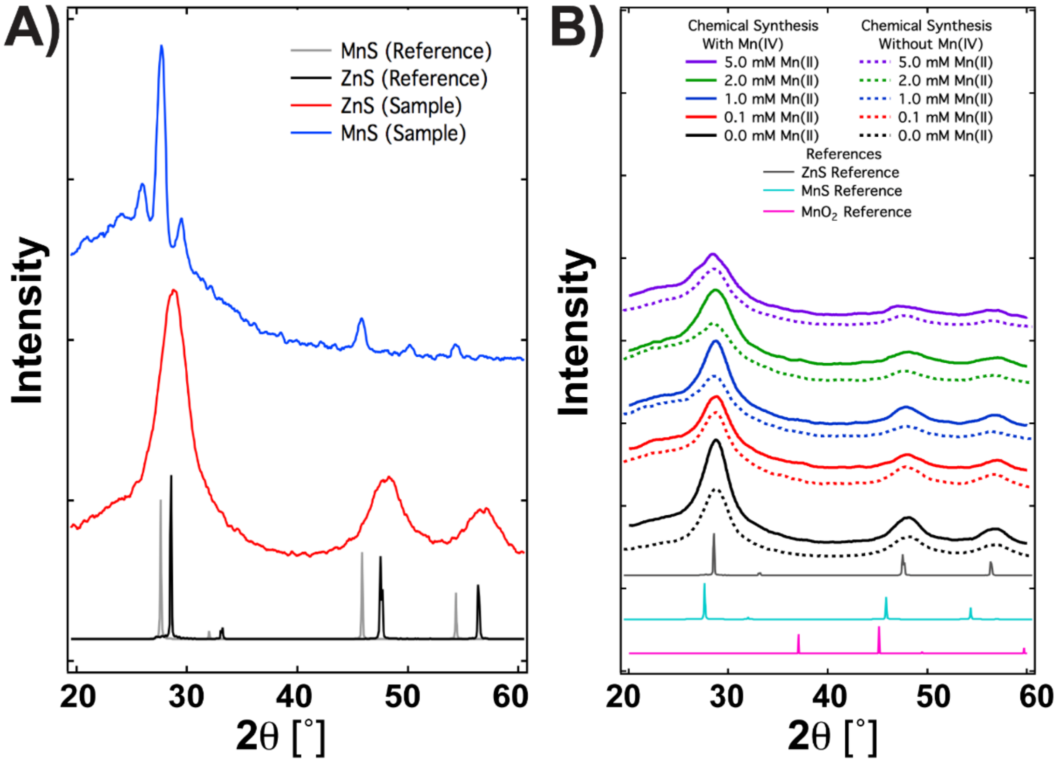

Figure S9: A) A comparison of of XRD patterns of chemically synthesized ZnS, MnS, and their respective references. MnS was synthesized by combining equimolar Mn(II) acetate and sodium sulfide at 2.5 mM in anoxic 7 mM HEPES. The reaction proceeded for 16 hours at room temperature, nanoparticles were washed five times in DI water. For XRD characterization, samples were dropcast on a glass slide and dried. B) A comparison of XRD patterns of chemical synthesis of ZnS and ZnS:Mn(II) in the absence (dashed lines) and presence (solid lines) of 750 µM Mn(IV) oxide.

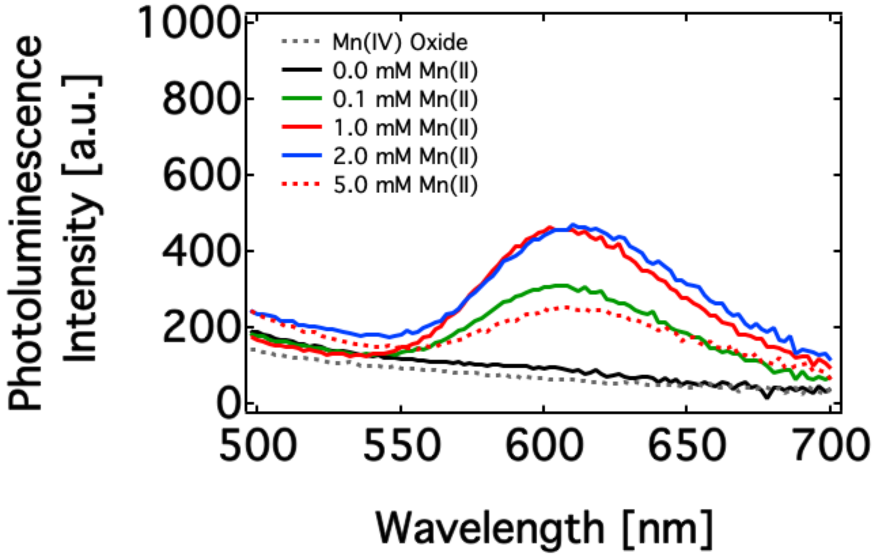

Figure S10: The influence of Mn(IV) on the chemical synthesis of ZnS:Mn(II) nanomaterial. The nanomaterial was chemically synthesized in the presence of 750 μM Mn(IV) oxide (added before zinc and sulfide addition) and variable amount of Mn(II). Nanoparticles of ZnS (for the case of 0 mM Mn(II)) or ZnS:Mn(II) formed, see Figure S4. Particles synthesized without the addition of Mn(II), Mn(IV) oxide only, did not show the characteristic photoluminescence peak around 600 nm.

Crystalline Domain Size	
Chemical Synthesis	
Mn(II) Concentration [mM]	Diameter [nm] (± 1.0 nm)
0	5.2
0.1	8.0
1	6.4
2	7.8
5	6.4
10	8.4
Biogenic Synthesis	
TMAO Concentration [mM]	Diameter [nm] (± 1.0 nm)
0.05	5.2
0.1	7.0
1.0	6.2

Table S2: Crystalline domain size as calculated from XRD data using the Scherrer equation. The error of ±1 nm comes from the uncertainty due to binning of the XRD data.

$$D = \frac{k\,\lambda}{FWHM\ \cos\theta}$$

Equation S1: Scherrer Equation: D is the domain size; *k* is a dimensionless shape factor that we set equal to 0.9; λ is the wavelength of x-ray, which is 0.154 nm in this experiment; the *FWHM* is the full-width at half-maximum; θ is the location of the maximum of the diffraction peak. We select the (111) peak at 28.7°

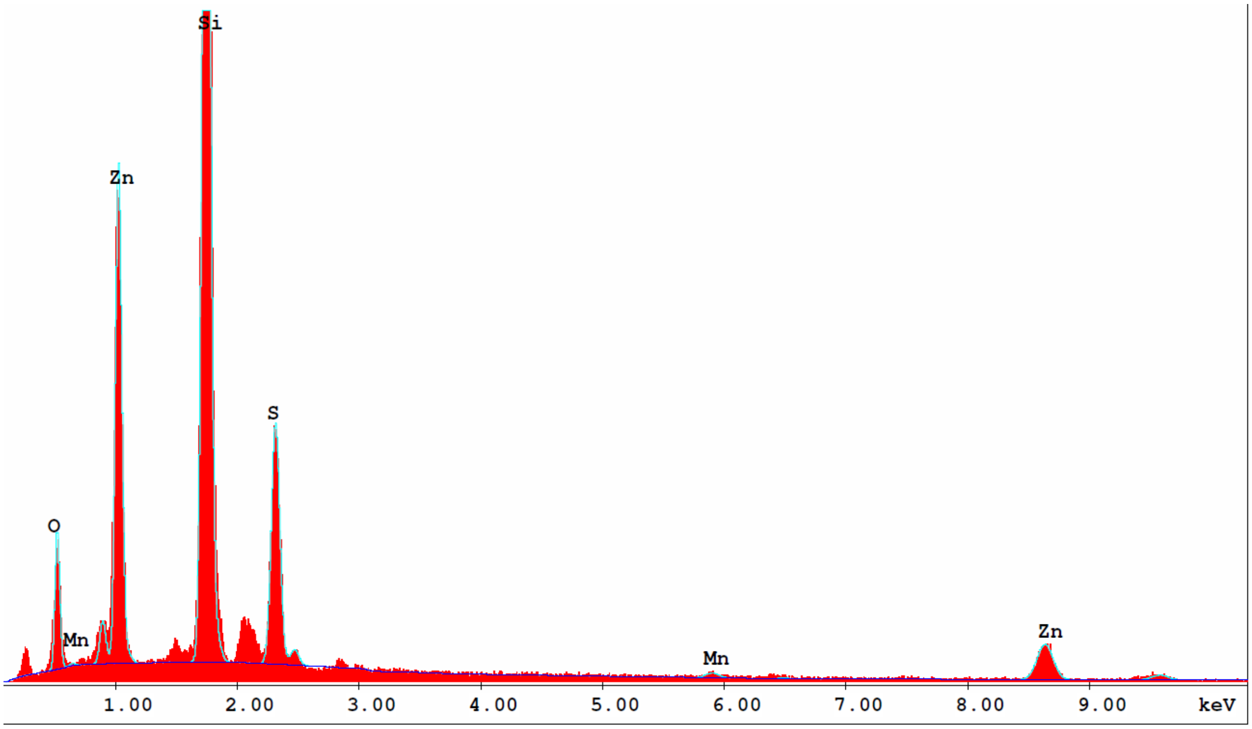

Figure S11. EDX Spectra for the ZnS nanomaterial synthesized by the chemical route with 2mM Mn(II)

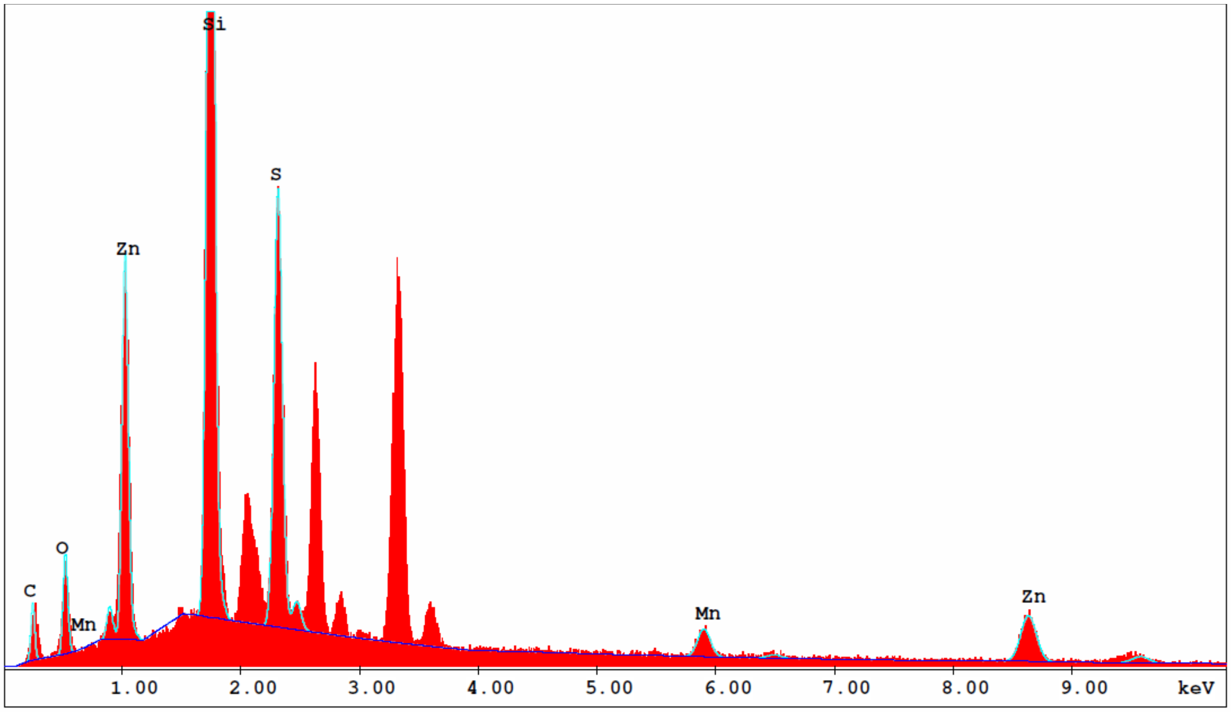

Figure S12. EDX Spectra for the ZnS nanomaterial synthesized by the biogenic route with 1 mM TMAO inducer molecule.

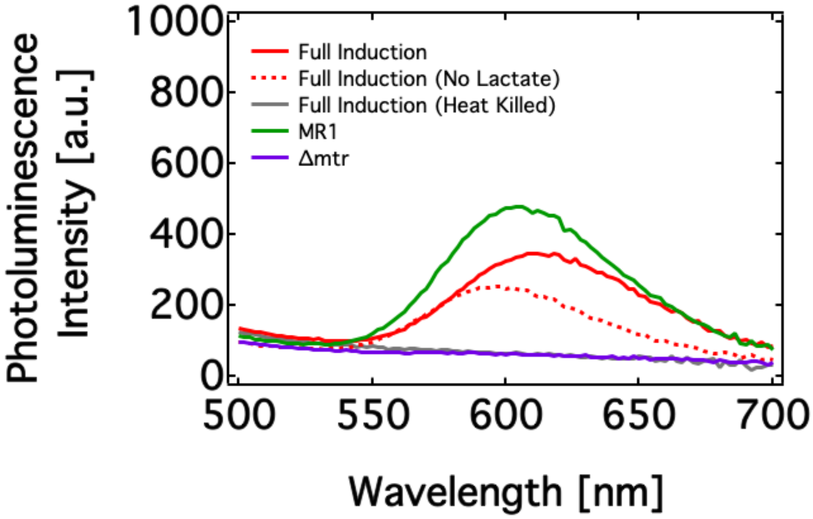

Figure S13: Influence of cellular activity on Mn doping. Nanoparticle synthesis was performed using heat killed cells JG3631, in cultures of JG3631 without a carbon source, and with the *mtr* deletion strain JG1486. To heat kill the cells, overnight cultures of fully induced cells (those incubated in minimal medium supplemented with 1 mM TMAO) were heated to 80˚C for 3 hours in a water bath. Plating confirmed that the density of live cells after the heat killing step was below 10 cells/mL (no colonies observed on the plates). Cells were heat killed prior to the Mn(IV) reduction step, and the standard procedure for biogenic synthesis described in the main text was followed. No lactate indicates that after the washing step, cells were resuspended in HEPES without lactate prior to the addition of Mn(IV). Wildtype *Shewanella oneidensis* MR1 and fully induced *Shewanella oneidensis* JG3631 are show as controls. Full induction indicates cells were cultured in the presence of 1 mM TMAO.
